# Outcomes and prognostic factors in childhood-onset steroid-resistant nephrotic syndrome

**DOI:** 10.1007/s00467-025-06977-x

**Published:** 2025-10-03

**Authors:** Emil Zerkowitz, Verena Klämbt

**Affiliations:** 1 Department of Pediatric Gastroenterology, Nephrology and Metabolic Diseases, Charité Universitätsmedizin Berlin, Berlin, Germany; 2 Berlin Institute of Health, BIH Charité Clinician Scientist Program, Berlin, Germany

To the Editors,

We thank Dr. Cheng and Dr. Liu for their interest [1] in our article on outcomes and prognostic factors in childhood-onset steroid-resistant nephrotic syndrome (SRNS) [2] and for their constructive comments highlighting the importance of careful statistical modelling. We welcome the opportunity to clarify our analytical approach.

While the 10 events per variable (EPV) rule is a useful guideline to reduce the risk of overfitting in observational studies with small sample sizes, it should not be regarded as an absolute threshold. Simulation studies by Vittinghoff and McCulloch [3] have demonstrated that logistic and Cox regression models can provide valid estimates with EPVs as low as 5–9, particularly when predictors are clinically justified and collinearity is limited.

Contrary to the impression that we included 12 predictors in the logistic regression for remission and 14 predictors in the Cox regression for kidney failure [1], our final models were more restricted [2]:

- Remission status: 38 events, 7 predictors (EPV 5.43).
- Follow-up analysis: 5 laboratory predictors for 38 events at 3 and 12 months (EPV 7.60).
- CKD stage V (Cox model): 4 baseline predictors for 23 events (EPV 5.75) and, separately, 5 follow-up predictors (EPV 4.60).

Predictors were selected based on clinical plausibility, prior statistical significance, and a transparent strategy to reduce dimensionality to mitigate overfitting.

Finally, as we emphasized in our paper, our analyses were exploratory and intended to identify potential prognostic factors, not to propose a definitive predictive model. We agree that validation in larger SRNS cohorts will be essential to confirm these findings.

## Data Availability

All data supporting the findings of our study are also available upon request.
